# Recycled aluminium cooking pots: a growing public health concern in poorly resourced countries

**DOI:** 10.1186/s12889-020-09485-9

**Published:** 2020-09-16

**Authors:** Angela Mathee, Renée Street

**Affiliations:** 1 Environment & Health Research Unit, South African Medical Research Council, Johannesburg, South Africa; 2 Department of Environmental Health, Faculty of Health Sciences, University of Johannesburg, Johannesburg, South Africa; 3 Department of Environmental Health, School of Behavioural and Lifestyle Sciences, Faculty of Health Sciences, Nelson Mandela University, Port Elizabeth, South Africa; 4 School of Nursing and Public Health, Discipline of Occupational and Environmental Health, University of KwaZulu Natal, Durban, South Africa

**Keywords:** Artisanal, Cookware, Aluminium, Lead, Exposure, Pots, Cottage industry, Informal

## Abstract

Lead exposure remains a significant public health problem, particularly in the informal sector. Recycling of scrap metal into artisanal pots is a growing concern in poorly resourced countries. Owing to the relatively light weight and low cost of the artisanal pots, as well as good conductivity which equates to lower usage of wood fuel, the pots are widely used. The aim of this article is to describe current insights and emerging evidence of health risks associated with artisanal pot making and usage. This thriving industry, particularly in poorly resourced communities, has multifaceted occupational, environmental and human health impacts. Given the complexity, innovative solutions need to be prioritized, evaluated and scaled up in relevant settings.

## Background

For decades lead exposure and poisoning have been considered high priority environmental health concerns in countries around the world. A recent study on four foodborne metals, namely mercury, lead, arsenic and cadmium, revealed that ingestion of these metals resulted in more than one million cases of ill health and more than 56,000 deaths. Of the cases of illness caused by these four metals, over half (54%) were due to lead, 22 and 20% from methylmercury and arsenic respectively and 1% from cadmium [1]. The growing body of evidence on the harms caused by lead, especially in children, sparked major protective interventions, predominantly in well-resourced countries. Similar lead poisoning prevention actions have been lagging in developing countries, despite lead exposure widely being regarded as more pervasive and serious in these settings. Of particular concern has been the extent of lead exposure emanating from the informal sector in developing countries, presenting public health and economic challenges that are particularly insidious and unyielding to environmental health action. Cottage industries are a group of informal income generating activities, mostly at individual, family or small group level, that take place within the home environment [2], such as electrical repairs, motor vehicle repairs, spray painting and artisanal pot making, many of which are associated with lead exposure. A particular concern with lead exposure from cottage industries is the potential for all family or household members to be exposed on a chronic basis [2]. The contribution of lead exposure, and the concomitant illnesses and burden of mortality, from cottage industry exposure as well as the use of resultant products such as artisanal cookware, has not been evaluated.

Aluminium is extensively used in the food industry and has been used to produce cookware and utensils since the late nineteenth century. Coating and anodization processes have rendered aluminium cookware surfaces relatively non-reactive, lowering the rate of leaching of the metal from cookware into foodstuffs. As a result, aluminium cookware, produced in the formal sector using standardized processes, is widely used by households around the world [3]. In some parts of the world however, the production of cookware from aluminium waste, using sub-standard processes, has been undertaken for decades, raising public health concern, especially with regard to chronic human exposure to contaminants such as lead and arsenic [4]. In order to determine effective ways to reduce exposure to toxic metals associated with artisanal cookware, there is a need to gain a deeper understanding of the processes involved, and assess and prioritize the risks to health of pot makers and users, as well as potential for contamination of the local environment. The aim of this article is to describe current insights and emerging evidence of health risks associated with artisanal pot making, which is thriving in some poorly resourced countries, and has multifaceted occupational, environmental and human health impacts.

## The contextual benefits and risks of using recycled Aluminium cookware

### Perceived advantages of artisanal Aluminium cookware

In settings of poverty, such as in many parts of Africa, artisanal aluminium pots crafted from waste metal have high appeal. They are relatively inexpensive, and their heating times are shorter, leading to decreased use of fuels such as coal and wood, which in some areas are becoming increasingly scarce. Artisanal aluminium pots are light in weight, and relatively easy to carry around, for example by children and women, who most frequently bear the burden of cooking and collection of wood fuel. The cost is low due to the ready availability of waste aluminium to produce cooking pots, and the manufacturing technology is relatively simple. Aluminium pots are also fairly strong, durable and resistant to corrosion [3].

### The production of artisanal Aluminium cookware in African settings

**Fig. 1:**
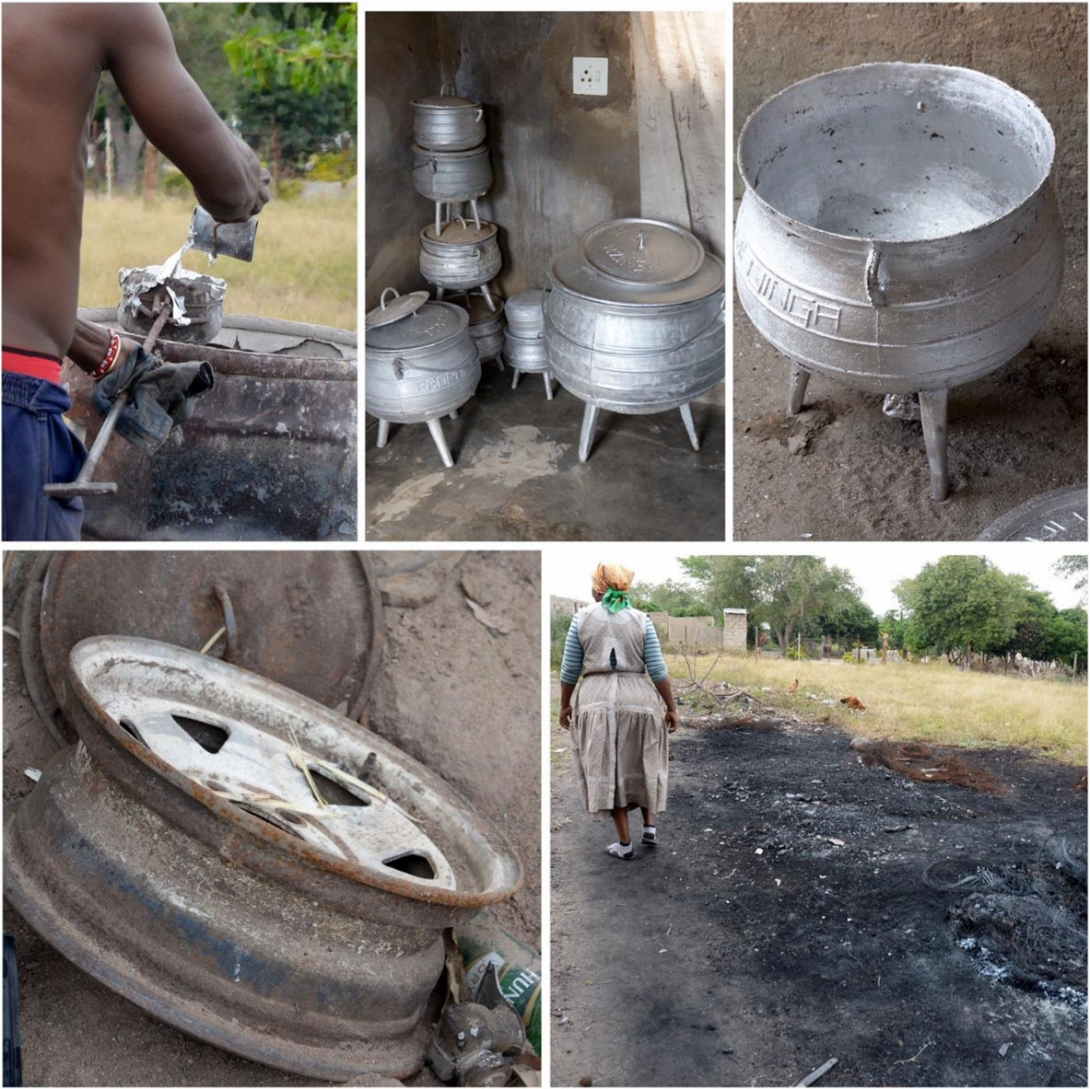
Images from an artisanal aluminium cottage industry in South Africa (Photographs by A. Mathee)

Artisanal aluminium cookware may be forged from a variety of waste metals, including motor car and bike engine parts, radiators, cans, construction materials and household appliances (Fig. 1) [4, 5]. The process of melting aluminium waste and crafting the pots is usually undertaken by a pair of individuals, or in small groups in a home setting [5, 6]. The work is laborious, and holds a risk of burn and other injuries, as well as exposure to air, dust and soil pollution. It may take as long as half a day for one or two men to make a single pot. The requirement for relatively low temperatures to melt the scrap aluminium has been associated with a high level of porosity in the final product. The non-standardization or subjectivity associated with the pot making process leads to a high defect rate, which may be costly for the pot makers in terms of both time and income. Field studies by the authors revealed that epoxy resins were sometimes used to fix small defects. Pots are often sold through direct sales or at local markets. In South Africa in recent years, there is anecdotal evidence that the production and use of artisanal pots is increasing, with the market growing from production and use in two provinces, to the current situation in which artisanal pots are available in at least six of South Africa’s nine provinces (Renee Street and Angela Mathee, personal communication). In parts of some African countries, the use of artisanal pots is pervasive, including in homes, schools, restaurants, for cooking of street foods and for catering at social gatherings, such as weddings and funerals, leading to a high potential for widespread and chronic exposure to lead and other toxic metals. Artisanal aluminium pots have also become a source of pride or considered a family heirloom, with names and signature designs being crafted onto the pots (Renée Street and Angela Mathee, personal communication) (Fig. 1).

### Studies on leaching of metals from artisanal Aluminium cookware

Despite the widespread artisanal production and use of artisanal aluminium cooking pots, until relatively recently there has been a paucity of research undertaken to assess the concomitant lead exposure through the food pathway. A paper from 2010 points to artisanal cookware as a potential source of lead exposure in 500 pregnant women from Bangladesh. Lead concentrations measured in erythrocytes, urine, and breast milk were found to be relatively high, and the authors suggested that cooking pots may be an important source. Preliminary investigations found that up to 380 μg/L of lead was released into acidic solutions from artisanal cookware [7]. In Uganda, Mbabazi and colleagues [8] found that the highest rate of leaching of aluminium was from pots that had been locally made by artisans, relative to pots of factory origin. In Thailand, the blood lead levels of children whose food had been cooked in inexpensive pots without a quality assurance certificate was significantly elevated relative to those eating from certified cookware [9].

In a more detailed study of artisanal aluminium cookware undertaken in Cameroon, analyses of 26 pots obtained from four regions showed that while the lead content of the pots was relatively low (< 1000 ppm by X-ray fluorescence [XRF]), significant amounts of aluminium, lead and cadmium were released during dilute acetic acid extractions through boiling, and standing at ambient temperatures (up to 899 μg of lead/L). Associated calculations indicated that the lead level per serving could be as high as 260 μg. The authors postulated that daily use of such artisanal cookware may lead to chronic exposure to lead exceeding international public health guidelines, with concomitant health risks for households, especially children [10]. The Cameroonian study was followed up with a more extensive investigation of the metal content of 42 locally made pots from ten countries (Bangladesh, Guatemala, India, Indonesia, Ivory Coast, Kenya, Nepal, Philippines, Tanzania and Vietnam). XRF analyses showed detectable levels of lead in 86% (36/42) of the cookware items, and 11/42 or 26% of the cookware items had a lead content exceeding 0.1% or 1000 ppm. Thirty six percent (15/42) of the pots released a lead concentration of > 1 μg per serving after boiling for 2 h with a dilute (4%) acetic acid solution. The leaching tests showed that up to 177 μg of lead was released per serving [4]. Most recently, in a South African study of 20 artisanal aluminium pots, lead was detected in all samples of leachate collected after pot sections held at 95 °C for 2 h in a 3% acetic acid solution. The mean concentrations of lead in leachate exceeded the European Union maximum permissible level of 10 μg/L in 11 (55%) of samples in the first of a series of three migration tests. For individual pots, mean lead concentrations in leachate ranged from 31 to 2050 ppm [5]. The first three above mentioned leaching tests were done using assimilated cooking (acetic acid). A further study of artisanal aluminium pots from four African countries assessed the impact of artisanal cookware on contamination of food samples. The study assessed Al, As, Cd, Hg, and Pb exposure when preparing tomato and the other staple foods in stainless-steel compared with artisanal aluminium cookware. Using a concentration factor (CF), which measured the ratio between the metal content in the identical sample prepared with aluminum vs. stainless-steel cookware, the magnitude of the migration of Al and Pb from artisanal aluminium was noteworthy. For example, the mean CF for Pb was 18 mg/kg, with the highest CF for Pb at 26 mg/kg which was from artisanal cookware from Cameroon [11].

## Discussion

The vast majority of children with elevated blood lead levels live in developing countries [12]. They are exposed to lead from a range of long-standing and emerging sources, emanating from the formal (regulated) and informal sectors [13]. Their elevated levels of lead exposure stymie children’s development, as well as the socio-economic prospects for families, communities and nations [12, 14–16]. The distribution of elevated blood lead levels is socially stratified, with poorer communities exhibiting significantly elevated burdens of lead exposure, which increases or entrenches social inequalities [16].

International efforts in recent years have catalyzed global controls on the use of lead in petrol (gasoline) and paint. However multiple sources of exposure to lead remain in poorly resourced countries, often within the informal sector. Cottage industries constitute a sub-category of the informal sector, in which the use of lead, and concomitant lead exposure, is particularly pernicious and intractable. A small number of studies undertaken during recent years have shone a light on potential lead exposure from the operation of small backyard foundries to smelt waste aluminium and forge artisanal pots [4–7, 10]. Concerns relate to lead exposure amongst cookware makers during the production process, end-users from contamination of cooked or stored food, and from lead contamination of the local environment.

## Conclusion

Addressing sources of lead exposure from the manufacturing and use of artisanal aluminium cookware is likely to be highly complex because of the relatively low cost of the cookware and lower usage of wood fuel, ease of use and the role of artisanal pots in the generation of household livelihoods. However, given the widespread and frequent use of artisanal pots in affected countries, likely constituting a chronic source of lead exposure to large numbers of people, and the concomitant impacts on public health, it is imperative that innovative solutions be prioritized, evaluated and scaled up as appropriate. With regard to research priorities, it is important to gain a deeper understanding of the extent of artisanal pot production in resource-poor countries, concomitant exposure to toxic metals amongst pot makers, their household members and consumers (including pregnant women and young children), the local environmental consequences of pot making and the costs and benefits of a range of protective interventions.

## Data Availability

Not applicable.

## Abbreviations

Al: Aluminium
As: Arsenic
Cd: Cadmium
Hg: Mercury
Pb: Lead
ppm: Parts per million
XRF: X-ray fluorescence
CF: Concentration factor
μg: Microgram
mg: Milligram
L: Litre
kg: Kilogram
